# Enhancing biomedical search interfaces with images

**DOI:** 10.1093/bioadv/vbad095

**Published:** 2023-07-17

**Authors:** Juan Trelles Trabucco, Cecilia Arighi, Hagit Shatkay, G Elisabeta Marai

**Affiliations:** Department of Computer Science, University of Illinois Chicago, Chicago, IL 60607, USA; Department of Computer and Information Science, University of Delaware, Newark, DE 19716, USA; Department of Computer and Information Science, University of Delaware, Newark, DE 19716, USA; Department of Computer Science, University of Illinois Chicago, Chicago, IL 60607, USA

## Abstract

**Motivation:**

Figures in biomedical papers communicate essential information with the potential to identify relevant documents in biomedical and clinical settings. However, academic search interfaces mainly search over text fields.

**Results:**

We describe a search system for biomedical documents that leverages image modalities and an existing index server. We integrate a problem-specific taxonomy of image modalities and image-based data into a custom search system. Our solution features a front-end interface to enhance classical document search results with image-related data, including page thumbnails, figures, captions and image-modality information. We demonstrate the system on a subset of the CORD-19 document collection. A quantitative evaluation demonstrates higher precision and recall for biomedical document retrieval. A qualitative evaluation with domain experts further highlights our solution’s benefits to biomedical search.

**Availability and implementation:**

A demonstration is available at https://runachay.evl.uic.edu/scholar. Our code and image models can be accessed via github.com/uic-evl/bio-search. The dataset is continuously expanded.

## 1 Introduction

Figures in biomedical papers convey evidence of discoveries, present results and outline experimental methods (Cohen *et al.*, 2003; Shatkay *et al.*, 2006; Yu *et al.*, 2009). These images also support identifying documents of interest in biomedical research and clinical practice (Li *et al.*, 2021). For example, clinicians interested in pulmonary fibrosis and its relationship with COVID-19 may wish to filter extensive collections, such as the CORD-19 collection provided by the Allen Institute (Wang *et al.*, 2020), for those papers containing ultrasound figures. The information retrieved can be relevant to patient cases, in particular in emerging situations where a population with the same characteristics is not readily available to clinicians.

Although searching for relevant publications is a prevalent research activity, most state-of-the-art search systems do not leverage publication figures. For example, both Google Scholar and PubMed Central (PMC), two of the most popular engines, only leverage text and citation data. A few systems support searching over images and captions separately (Demner-Fushman *et al.*, 2012; Divoli *et al.*, 2010; Lee *et al.*, 2017), but provide limited support for filtering by image-derived data. Likewise, Google Images allows faceted search by classifying some types of biomedical images, but Google Scholar lacks this feature. Similarly, searching over captions and full text improves retrieval (Divoli *et al.*, 2010), but we do not know whether presenting image data in publication search supports identifying relevant content.

Several challenges hinder the incorporation of images into academic paper searches. First, a lack of understanding of which image data enhances document retrieval can drive efforts in the wrong direction. For instance, content-based image retrieval, as provided by Google Images, requires an input figure, but in biomedical domains like biocuration, targeting one image at a time is counterproductive. Alternatively, categorizing the image data into meaningful imaging modality categories improves identifying relevant documents for the biocuration of mice gene expression (Li *et al.*, 2021; Shatkay *et al.*, 2006). However, while there is work in categorizing general medical images (Demner-Fushman *et al.*, 2012; Lee *et al.*, 2017), there is no work organizing biomodalities into a taxonomy meaningful to, e.g. COVID-19 biocuration.

The second set of challenges concerns the efforts required to integrate such data and taxonomy. Since figures reside inside the PDF documents, preprocessing tools need to extract the figures and identify regions to annotate based on taxonomic categories. Even if future scientific publication standards will require authors to separately upload each paper figure, there are millions of existing documents that will still require preprocessing. Consequently, the image data needs to be automatically extracted, identified and tagged according to the proposed taxonomy. This requirement argues for models to correctly tag the figure content.

There are also many challenges related to designing and evaluating a search system to support figures. On the front end, complex user interface designs can affect negatively the search experience (Hearst, 2009). Existing search solutions range from showing images in isolation to showing text data alone, but not both. In addition, while several studies analyzed the integration of images and text for simple web search (Capra *et al.*, 2013; Dziadosz and Chandrasekar, 2002), few studies (Divoli *et al.*, 2010) evaluate the presentation of results for academic search. On the back end, a scalable system would need to manage indexes, store database records and image content and provide scalable interfaces to access such data.

In this work, we describe a search system for biomedical documents that leverages image modalities as a proxy for image content. These image modalities are organized in a COVID-19 taxonomy that we derived and validated through a multi-year collaboration with biocurators. The modalities describe a figure’s acquisition methods, e.g. whether the image was generated by a microscope. We then describe the design and implementation of such a search system, from integrating a document preprocessing pipeline, to document indexing, and to presentation. Overall, we combine into a novel product existing extraction and classification techniques. We furthermore propose an innovative search interface to support the integration of figure modality information. We report the usability results of a formal evaluation with biomedical researchers. Last but not least, we report lessons learned and validate the efficiency of the components of our system.

## 2 Methods

Academic search systems index diverse fields, such as titles, abstracts, captions, and even the full text to achieve their retrieval goals. On the front end, user interfaces show this content using summaries of a search result (i.e. document surrogate), which display data from the document and explain why it was retrieved (Hearst, 2009). This section explains how we integrate image modalities into document search and expand document surrogates with image content. The project was developed over 3 years, from the taxonomy derivation to the interface design, testing and evaluation.

### 2.1 Taxonomy of image modalities and models

In order to tackle figure information, search systems mainly use two categories of figure surrogates. In the first approach, the figures and the captions are extracted and displayed in brick-wall layouts (Demner-Fushman *et al.*, 2012; Lee *et al.*, 2017) or in grid patterns (Divoli *et al.*, 2010). The document information is not shown unless available on demand. In the second approach, hybrid document surrogates consisting of figures and basic document metadata are shown next to each other, displaying either all figures in the document (Divoli *et al.*, 2010; Lee *et al.*, 2017) or displaying only one figure at a time (Demner-Fushman *et al.*, 2012). While recent approaches use image modalities as search filters (Demner-Fushman *et al.*, 2012; Lee *et al.*, 2017), these systems do not identify directly the location of images.

In practice, a publication figure can contain many subfigures (Li *et al.*, 2021). Moreover, each subfigure can belong to a different image modality or submodality. Subcategories can, in turn, influence the document retrieval results. For example, grouping images only in coarse categories [e.g. considering radiology and microscopy images as photographs (Lee *et al.*, 2017)] does not allow users to distinguish between radiology and microscopy. Taxonomies are thus necessary in order to properly identify and tag subfigures into meaningful subcategories. In fact, results from document classification experiments show that such taxonomies serve as an essential proxy for estimating document relevance in biocuration (Li *et al.*, 2021; Shatkay *et al.*, 2006).

This work leverages a taxonomy initially developed to help label image modalities in the CORD-19 dataset (Trelles *et al.*, 2021). Our interdisciplinary group features expertise from biocuration, text-mining and visual computing researchers at three institutions with experience in developing biocuration tools (García Seco de Herrera *et al.*, 2016; Shatkay *et al.*, 2006): the Protein Information Resource at the University of Delaware, WormBase at the California Institute of Technology and the University of Illinois Chicago. We derived this taxonomy starting from an existing ImageCLEF taxonomy (García Seco de Herrera *et al.*, 2016), and expanded and further subdivided categories via monthly meetings and discussions over 2 years. Using this expert-provided taxonomy to label biomedical images is essential in biocuration because it provides the necessary high-level abstraction of the image content and cues for document relevance. We validated the evolving taxonomy through several design iterations of a labeling tool and repeated application to the images in the CORD-19 dataset. This process also yielded a set of labeled images, which we then used to train classifiers for tagging other image data.

**Figure 1.**
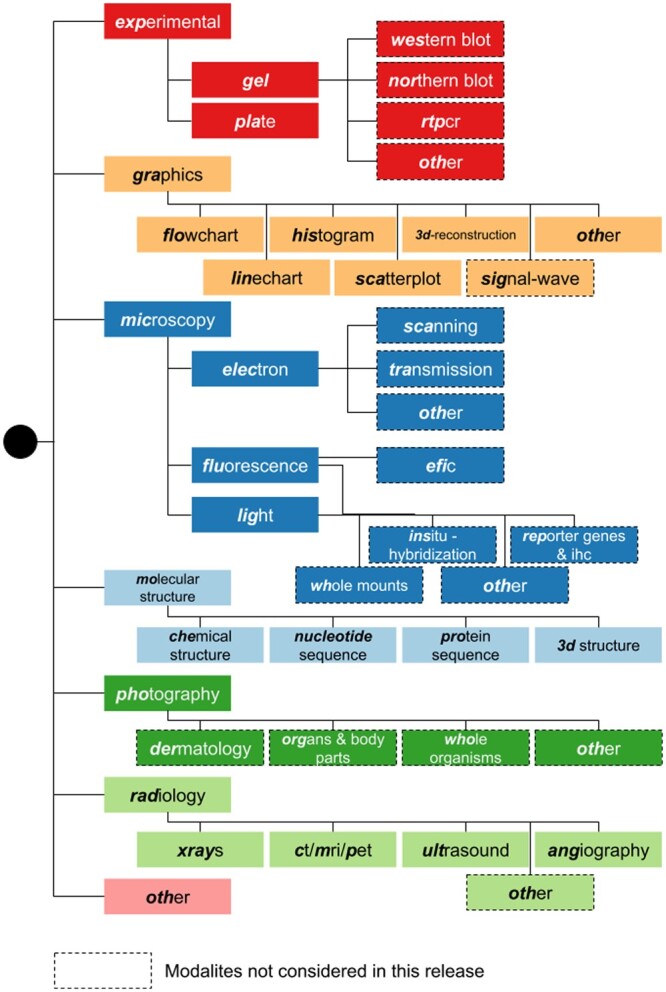
Taxonomy of modalities derived for a collection of COVID-19 papers. Dashed lines indicate modalities not included in the current release of the search system. Colors differentiate each modality branch

The resulting taxonomy (Fig. 1) includes seven main categories: experimental, graphics, microscopy, molecular structure, photography, radiology and others. The experimental category encompasses gels and plates. We further divided gels into western blots, northern blots, RT–PCRs and others. The graphics category includes flowcharts, histograms (and bar-based charts), line charts, scatterplots, signals/waves, 3D reconstructions and others. The microscopy category comprises light, fluorescence, and electron microscopy. Subcategories in light and fluorescence microscopy include experimental methods like *in situ* hybridization, reporter genes & immunohistochemistry, whole mounts and others. For fluorescence microscopy, we also include the EFIC method. The electron microscopy class includes scanning, transmission and other subcategories. The molecular structure category comprises chemical structures, 3D structures and nucleotides & protein sequences. Photography includes dermatology, organs & body parts, whole organisms and others. Finally, radiology includes X-ray, CT-scan/MRI/PET, ultrasound, angiography and others. The search system leverages only the first two taxonomy levels due to the current scarcity of labeled samples at lower levels.

### 2.2 System architecture

**Figure 2.**
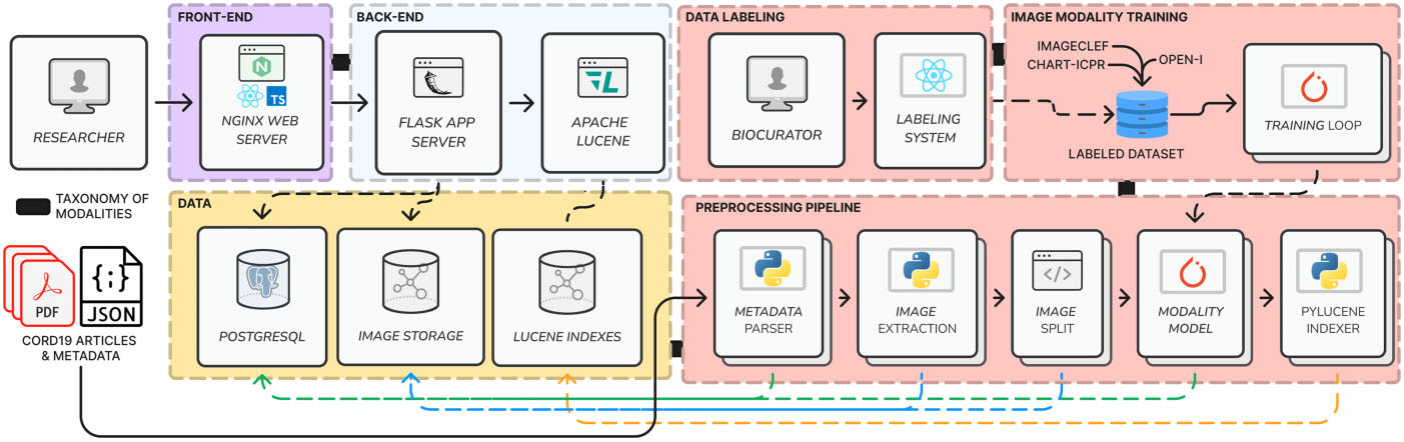
The system architecture. The online components include a web interface, an application server to retrieve data from local storage, and an Apache Lucene search engine to process queries. The offline components are responsible for labeling, model training and document preprocessing

The architecture consists of online components to process queries, and offline components to parse document data (Fig. 2). The front end uses a web interface developed in React, hosted in an Nginx web server. It communicates with a Flask server, which accesses the content stored in a PostgreSQL database (metadata) and local storage (image content). It also interacts with an Apache Lucene engine to delegate queries.

**Figure 3.**
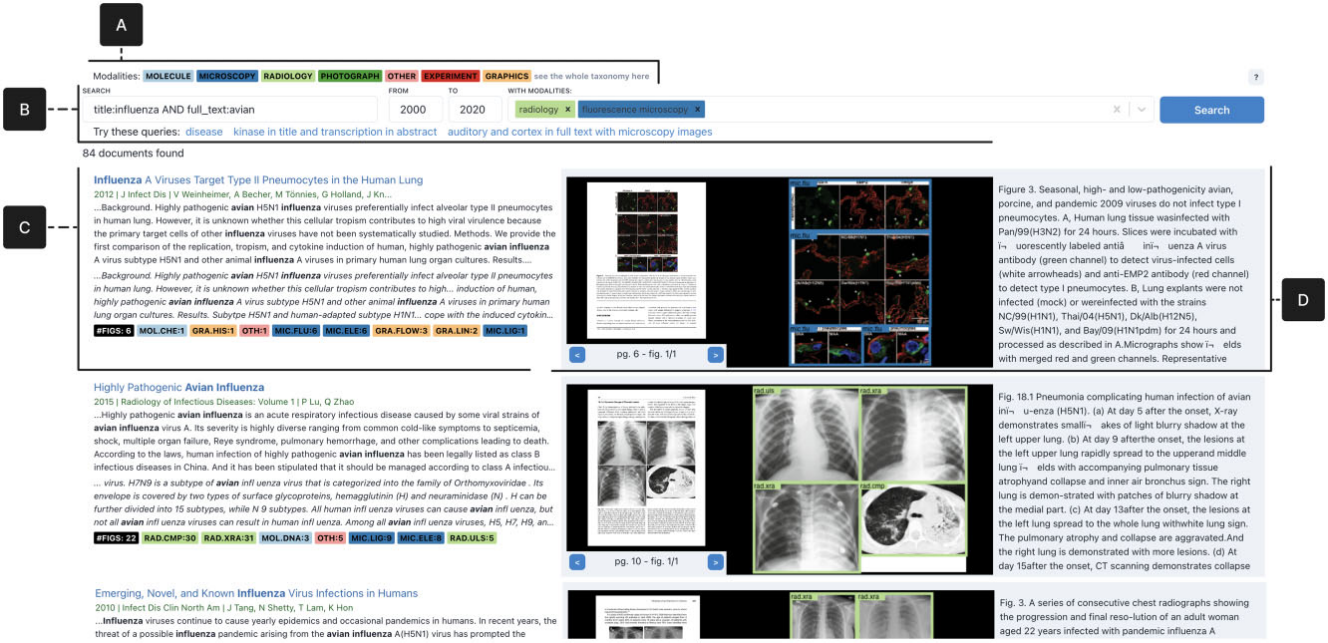
Search interface displaying the results of a Boolean query. (**A**) The color-coded legend displays the image modalities in the first level of the taxonomy. (**B**) The search bar, with a keyword box showing a Boolean query, a time filter and an image modalities filter limiting results to documents containing *radiology* or *fluorescence microscopy* figures. (**C**) The metadata of a result includes the document title, year, venue, authors and the hits on the abstract and full text. Below is the count of figures and modalities in the document. (**D**) The enhanced document surrogate shows the image content, including the page thumbnail, extracted figure, subfigures and modalities and caption

Because we aim to integrate a taxonomy to index figure data, offline components take the taxonomy as input and connect such taxonomy definitions across our solution. On the back end, we train image classification models. The preprocessing pipeline uses these models and state-of-the-art image processing tools to transform the input PDFs and metadata into database records, figures, subfigures, modality predictions and search indexes. The taxonomy of modalities acts as a connecting tissue between these components by setting the classifier classes and available indexes.

### 2.3 Dataset preprocessing

The dataset comprises a subset of documents from the January 2021 release of the Allen Institute CORD-19 collection (Wang *et al.*, 2020), which matched the project target audience of clinicians and biomedical researchers. The CORD-19 metadata includes information for document surrogates, such as title, publication year, abstracts, authors, journal and abstract. Because the PDF documents, and thus, the figures, were not available as part of the release, we limited the set to CORD-19 publications also indexed in the PMC open-access repository. We used the PMC FTP service to download 12 708 documents.

Our preprocessing pipeline comprises document and image processing tools (Fig. 2). First, we used PDFigCapX (Li *et al.*, 2019) to extract each figure from the PDF and match it with each caption. Then, we processed the figures with FigSplit (Li *et al.*, 2018) to find the constituent subfigures. These two tools have reported state-of-the-art results in extraction and segmentation tasks for biomedical documents. Next, we used image classification models customized to our taxonomy to identify the image modality. Finally, we indexed the document and modality information. At each step, we populated the database with the metadata from the processed data, including bounding boxes for figures, subfigures, modality predictions and prediction probabilities. Furthermore, we stored local copies of the figures and subfigures in a network drive.

We modeled our image classification components as ResNet18 convolutional networks, given its competitive performance compared to larger ResNet (He *et al.*, 2016) architectures. To train the models, we used biomedical images from the ImageCLEF13&16 subfigure classification task (García Seco de Herrera *et al.*, 2016). We further used chart images from CHART-ICPR-2020 (Adobe Research, 2019), primarily synthetic samples. Next, our biocurator collaborators helped us generate the remaining labeled images: ∼15 500 images predicted as gels or plates by an older classifier, around 2000 images collected and manually labeled from Open-I and around 6000 images labeled by biocurators (Trelles *et al.*, 2021) (Fig. 2, top right). Finally, we matched every label to match our taxonomy, and validated the match through sampling.

Because the labeled data did not completely match our taxonomy hierarchy (e.g. we knew some images were from the microscopy modality but did not know the microscopy submodality), we trained a collection of classifiers targeting each parent node in the taxonomy; thus building a hierarchy of image classifiers. This approach allowed us to use images with complete labels on models with sub-classes (e.g. gel classifier) and images with incomplete labels on parent classifiers (e.g. experimental classifier). To keep the data consistent across training, validation and test sets, we started dividing the subsets in a stratified manner bottom-up. As a consequence, an image must pass through several classifiers to get submodality details. We trained these classifiers using a 70/10/20 partition, a learning rate of 1e−4, and early stopping. We implemented these models using Pytorch.

### 2.4 Indexing

We used Apache Lucene for the search engine, and integrated our front and back end using the PyLucene python library. For each document, we indexed the title, abstract, publication date, journal, authors, DOI, pmcid, number of figures, full text and a list of all the modalities within the document. Following expert feedback during the evaluation, we also indexed figure captions. Although we do not currently search over all fields, we indexed the document fields for convenience during retrieval. In addition, we did not have weights for any indexed fields and thus used Lucene’s default scoring model, which weighs each query term equally. While the Lucene server provided the information for surrogates, we stored the image information on a PostgreSQL database. Our API allowed retrieving the location of every image extracted from the document, the bounding boxes for each subfigure and the predicted modality. Along with the image, we returned the source page thumbnail, an artifact created by our image extraction module. These image elements are used to enhance surrogates.

### 2.5 Interface design

We derived our interface design through several discussion meetings and testing sessions with collaborators, and we incorporated feedback from domain experts. Our top design includes a search panel, and a results panel with a card design for figures (Fig. 3).

To keep the design simple and consistent with the users’ previous experiences, our interface design emulates existing popular interfaces, such as Google Scholar and PMC. Our design adds modality information to the document surrogate and extends the search bar to allow filtering by modalities. Also, the search box allows users to input more complex Boolean expressions, although it does not include a faceted search.

To support learnability and minimize errors, we rely on consistent color coding, example queries, and detailed instructions available on demand. We provide visual feedback and text highlighting to familiarize users with the enhanced document surrogates, and to help them infer result relevance without needing to open an additional link. When a modality filter is specified, we rank the figures in the document to prioritize the images containing the larger number of subfigures of the requested modality. Finally, following lessons from web search (Aula *et al.*, 2010), result cards have a height of 300 px to avoid harming the visual content.

In terms of the document surrogate information shown, while the proposed card designs varied between design iterations, every prototype card included a document page thumbnail, at least one extracted figure from that page and the corresponding caption. These three elements per card provided, according to the tester feedback, focus and context. During a first iteration, we discarded the idea of stacking the cards visually because the arrangement wasted space. Also, we noticed that displaying separate subfigures was less desired than showing bounding boxes over the original figure. Alternative designs included an embedded PDF page reader. However, we discarded the idea after discussion, and prioritized showing results in the list format more familiar to our collaborators, and targeting the relevance prediction task.

#### 2.5.1 Search bar

The search bar is the entry point to the system. On the left side, the search box allows performing a keyword-based search on the text fields: titles, abstracts, captions and full text. Simple keywords (e.g. *avian influenza*) trigger searches in any of the text fields using an OR operator. Words can be encased in apostrophes to perform exact matching. Key-value pairs following the syntax *<title*|*abstract*|*full_text*|*caption: keyword>* limit the search to specific fields. For instance, *title: influenza* triggers a search for the keyword *influenza* on the title field. Boolean operators compose more than one key-value pair with AND or OR operators. The remaining two filters limit the results by publication year and modalities in the paper. Above the search bar, the interface lists the categories from the first level of the taxonomy and their corresponding colors as a legend. The whole taxonomy can be displayed as details on demand.

#### 2.5.2 Search results

The search results follow a vertical list layout, where each row contains an enhanced document surrogate. Up to 10 results are shown per page of results, and pagination options at the bottom can be used to inspect more results.

On the left of each result panel, a simple document surrogate shows the title, year of publication and the query hits on the abstract and full text with term highlighting. We limit the number of matching lines to three and five, respectively. Below, a black badge indicates the number of figures in the document, followed by color-coded badges indicating the type of modalities found and the number of subfigures per modality.

The image content appears to the right. We show the document page thumbnail, one extracted figure and the caption if it exists. Also, we highlight matching terms in the captions. Overlay color frames surround the subfigures identified in the preprocessing steps, and we show their predicted and color-coded image modality. Hovering over the figure scales the content for better visibility. Controls support navigating between the rest of the figures, subfigures and corresponding page thumbnails. By default, figures are displayed in their order of appearance in the document. However, when users apply modality filters, the interface sorts the figures based on the count of subfigures matching, per figure, the specified modality.

### 2.6 Experiments and results

We evaluate the system’s performance and usefulness through a quantitative and qualitative approach. First, we perform a quantitative evaluation of the image classifiers, to estimate the performance of the system’s modality predictions during retrieval, when the retrieved images would not be pre-labeled. Second, we perform a quantitative evaluation of the system retrieval results against text-only retrieval. Finally, we perform a quantitative and qualitative evaluation of the system capabilities with nine researchers.

### 2.7 Image classifiers

To account for the quality of the modality predictions, we report first the performance of the classifiers used in the preprocessing pipeline, on the training, validation and test labeled data (70/10/20). Table 1 shows the accuracy and *F*1 scores for the validation and test sets for the parent node in the taxonomy (higher-modality), and for each of the classifiers at the first level of the taxonomy: experimental, graphics, microscopy, molecular, radiology and others. In addition, we show the number of labeled images used for training each classifier, to illustrate the impact of these numbers on the classifier performance. The rightmost column displays the number of predictions made on the unlabeled images extracted from the CORD-19 subset and indexed in our system. Accuracy and *F*1 scores are calculated as: Accuracy=(TP+TN)/(TP+TN+FP+FN) and F1=2×TP/(2TP+FP+FN), where TP, FP, TN and FN are true/false positive/negative.

**Table 1.** Classifier performance along with the number of labeled images used for training, validation and testing, and the number of image label predictions generated for the search system

	Validation		Test		Number of images	
Classifier	Accuracy	*F*1	Accuracy	*F*1	Labeled^a^	Predicted
Higher-modality	98.77	98.60	98.79	98.63	333 998	199 168
Experimental	98.92	98.89	98.98	98.97	23 873	28 754
Graphics	99.48	99.48	99.46	99.46	241 075	260 896
Microscopy	93.60	93.71	94.20	94.20	11 831	20 705
Molecular	95.20	95.23	92.81	92.82	1170	9306
Radiology	93.64	93.31	95.76	95.70	3023	4468

*Note*: The higher-modality classifier accounts for the top classes in the hierarchy.

a Total number of images in the training, validation, and test sets.

### 2.8 Retrieval results

Due to the lack of ground-truth retrieval data for this problem, and the fact that search scenarios suffer from computational complexity due to the vast list of possible use case scenarios (‘one cannot enumerate the other scenarios’), we focused on a number of scenarios generated by biocurators with the explicit aim of creating sufficient and representative coverage of the query space. Some of the scenarios target the COVID-19 problem, and some target more general problems. The inputs were Boolean queries over the full text searching for keywords like chest, or kidney inflammation. The first scenario looked for (i) *COVID-19* papers with the *chest* keyword and *radiology* images, (ii) then restricted the modality to only one radiology submodality: *x-ray*. The next scenario (iii) looked for *avian influenza* papers with *CT scans*, followed by a search for (iv) *interactomes* and *blots* (a proxy for *experimental gel* images). Finally, we looked for (v) *kidney inflammation* papers that included *microscopy* images.

Each scenario consisted of two conditions aiming to retrieve documents containing images of particular modalities. The first condition (text-only) used keywords only to filter the content and one keyword as a proxy for the image modality. The second condition (text+image) used the exact content keywords but included a modality filter based on our taxonomy. The results were manually inspected and reviewed during several team meetings to calculate the number of relevant documents. We report the number of documents retrieved (documents retrieved by both the text-only and text-image queries), the overlap between the two sets of retrieved documents, the precision and recall and the number of images returned for each condition, where: Precision=TP/(TP+FP) and Recall = TP/(TP+FN), where TP, FP and FN are relevant/non-relevant documents retrieved/not retrieved.

Table 2 shows the retrieval results for text-only and text+ image queries for each scenario. In Scenario 1, the text+image query retrieves all the relevant documents (13/13), while the text-only query retrieves less than a third (4/13) of the relevant documents (recall 0.31 compared to 1.0 for text+image). In Scenario 2, the text+image query retrieves all (7/7) of the relevant documents. In contrast, the text-only query also retrieves seven documents, but only three of those are relevant, leading to worse text-only recall and precision. In Scenario 3, the text-only query retrieves more documents than the text+image query (116 versus 47), but most of those documents are not relevant (precision 0.08), whereas the text+image query retrieves most of the relevant documents (recall 0.91). In Scenario 4, the text-only query retrieves fewer documents than the text+image query, and a larger number of relevant documents (precision 0.79 versus 0.60). However, the text+image query retrieves all the relevant documents compared to the text+image query (recall 1.0 versus 0.68). Finally, in Scenario 5, the text-only retrieves again fewer documents (191) than the text+image query (340), but the text+image query provides a higher precision (0.80 versus 0.63) and recall (0.99 versus 0.44). The higher recall in each text+image scenario shows that modality tags support the retrieval of relevant documents.

**Table 2.** Retrieval results

	Documents		Text-only				Text+image			
Scenario	Relevant	Overlap	Docs	Prec.	Rec.	Images	Docs	Prec.	Rec.	Images
1. COVID19 lung radiology	13	4	4	**1.00**	0.31	19	13	**1.00**	**1.00**	50
2. COVID19 lung x-ray	7	3	7	0.43	0.43	7	7	**1.00**	**1.00**	14
3. Avian influenza CT	11	11	116	0.08	0.82	55	47	**0.21**	**0.91**	106
4. Interactome blot	28	21	24	**0.79**	0.68	197	47	0.60	**1.00**	316
5. Kidney inflammation microscopy	274	130	191	0.63	0.44	1722	340	**0.80**	**0.99**	1029

*Note*: Best precision and recall scores are bolded. Relevant documents were counted manually.

Overall, for most scenarios, both the text-only and the text+image queries returned more documents than the total number of relevant documents, reflected in the precision scores. In particular, the avian influenza query, for both conditions, retrieved mostly non-relevant documents. Analyzing the overlap between the results under the two conditions provides further insight. Scenarios 3 and 4 capture minor differences in the total number of relevant documents retrieved in the two conditions (11/11 and 21/28, respectively). In contrast, text-only retrieves less than half the relevant documents in Scenario 1 (4/13), Scenario 2 (3/7) and Scenario 5 (130/274).

Upon further analysis, we found that the lower precision (0.21) for the text+image query in Scenario 3 is due to several grayscale images erroneously predicted as CT scans. The precision is still much higher than the text-only query (0.08); the recall is still 0.91. The lower precision (0.6 text+image compared to 0.79 text-only) in Scenario 4 is, likewise, due to several images mispredicted as gels, which increased the number of false positives. Finally, text+image queries harvested significantly more figures in every scenario than in the text-only condition. These results were expected because the figures matched the specified modality filter, although mispredictions may lead to higher numbers.

### 2.9 Expert evaluation

We asked nine researchers with significant biomedical expertise to evaluate the system. Four of the nine researchers are senior biocurators at North American institutions, three are biomedical researchers at different US institutions (e.g. cancer treatment centers), and two are senior doctoral researchers at a US institution. We did not collect any personal data or further information about the evaluators.

The evaluation protocol consisted of four steps, inspired by the evaluation structure proposed at the Bio-Creative VII demonstration for COVID-19 tools (Chatr-Aryamontri *et al.*, 2022). First, participants were required to read a brief introduction tutorial. Next, a guided activity listed a set of actions, to highlight the differences between text-only and text+image queries, and to introduce Boolean queries. Participants were directed to assess the system’s potential instead of minor limitations, such as occasional errors in figure extraction or mispredictions. Then, the exploratory activity required participants to try their own queries. For this exploration and the previous guided activity, participants were instructed to take notes to document their query experience.

The last step consisted of one questionnaire divided into three sections. The first section aimed to collect feedback, as well as suggestions for improvement. In accordance with the system usability score (SUS) (Brooke, 1996) guidelines, the following section provided 10 questions on a Likert scale (1–5), and one follow-up question on a scale (1–10). The third section asked seven additional questions, on a Likert scale (1–5), to further quantify the usefulness of specific interface elements (e.g. showing thumbnails).

Overall, the participants expressed high satisfaction with the system response to their queries. The resulting usability score (SUS score) was 87.22, equivalent to a grade of A, Excellent (>80.3). Participants agreed that they would use the system frequently (4.25/5), that the system was easy to use (4.75/5), and that it provided a low learning curve (4.87/5). Likewise, they did not find the system unnecessarily complex (1.5/5) and did not think they needed support from a developer to use the system (1.88/5). In addition, most expectations were satisfied (4.25/5), and they felt confident using the system (4.37/5). Overall, most participants would recommend the system to colleagues performing COVID-19 or biomedical related research (8.88/10), and participants agreed that the system supported their expectations (4.22/5).

Most participants (6/9) liked best the capability of seeing the document figures next to the search results. Some participants liked filtering papers by modality (experimental evidence) the most (3/9), and all participants praised its ease of use. One participant commented: ‘PubMed and Google Scholar do not display figures like this (system). Oftentimes all I do is scan the figures. This quick way to peruse articles is a huge improvement’. One participant suggested applying our pipeline to all open-access corpora.

While participants found the system functions well integrated (4.12/5), they also included several points for improvement. Three participants suggested indexing captions and highlighting matching terms on them. In particular, one participant identified a helpful use case for her workflow: ‘I have a list of genes whose expression pattern has not been curated in the reference database. I want to browse the literature (images) to see mentions of that gene in the figure caption’. The current version of our system now supports this requirement. Three participants requested displaying zoomed images with higher resolution, possibly on a separate panel. Three participants wished to export data from the system, including downloading citations, the list of results (including PMIDs), or even the full papers for further annotations. One participant wanted to copy figures for work-related presentations. Furthermore, two participants wanted to sort results by publication date. Other minor suggestions referred to various pipeline components.

**Figure 4.**
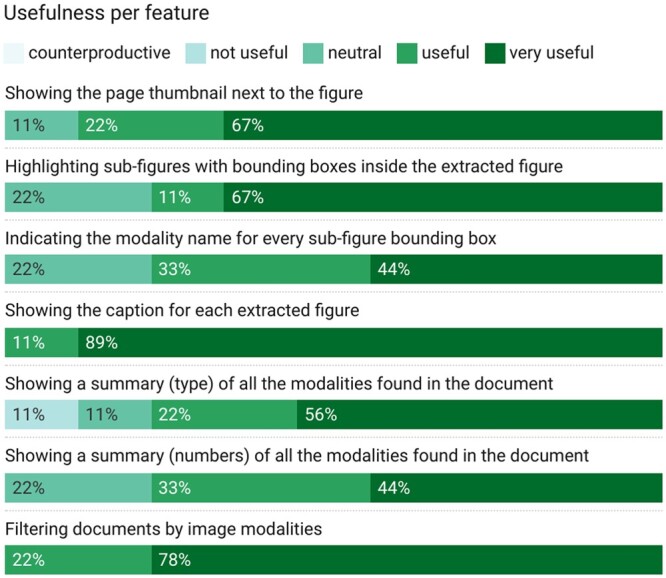
Results from the usefulness per feature section of the questionnaire, expressed in percentages

Figure 4 shows the results for the usability questions by feature. All participants considered showing captions and filtering by image modalities very useful features. Most participants further considered all features as very useful. However, one user found the count of modalities counterproductive. Likewise, one participant found the bounding boxes for subfigures not useful. This last opinion contrasted with another participant who listed the bounding boxes as the feature he liked the most.

## 3 Discussion and conclusion

Interacting with academic search interfaces is a prevalent research activity. Many search systems support rich query formulations on full text and faceted filtering on document metadata. In addition, search results appear in vertical lists of cards that display metadata and highlight sentences matching the input queries. However, while most academic websites like Google Scholar, PubMed, IEEE Xplore, ACM Digital Library and Semantic Scholar adopt this design, none display or provide alternatives to search for images within the documents based on modalities, as we do.

The evaluation shows that our solution effectively leverages the figure content to improve searching for relevant biomedical papers. We successfully demonstrated that categorizing the image data into meaningful imaging modality categories yields good results. The quantitative evaluation of the taxonomy classifiers yielded excellent accuracy and *F*1 scores, indicating the strengths of the taxonomy approach. The quantitative evaluation against text-only search further demonstrates that this text+image approach yields stronger results in terms of document retrieval precision and recall. Lastly, the excellent feedback from domain experts further highlights the benefits of including figures in the search. The high scores regarding potential adoption and frequency of use, and regarding recommending the system to other peers are particularly important measures of the potential of this approach.

Our solution successfully met the challenge of incorporating publication image data into queries. Pioneer systems [BioText (Divoli *et al.*, 2010) and Yale Image Finder (Xu *et al.*, 2008)] have searched only captions. Several other interfaces (Demner-Fushman *et al.*, 2012; Deng *et al.*, 2022; Lee *et al.*, 2017) use a variety of taxonomies to refine the same strategy. Compared to searching over captions, using more complex and powerful image-modality information requires developing a relevant taxonomy, extracting images from PDF documents, identifying subfigures and developing models to predict modalities, as we do.

Our solution also met a number of challenges in designing the front end for such a search system. The questionnaire feedback indicates we succeeded in adding significantly more visual content than other search interfaces, without creating friction in the user experience (Hearst, 2009). Our enhanced document surrogates differ from similar search interfaces. Compared to Viziometrics (Lee *et al.*, 2017) and BioText (Divoli *et al.*, 2010), we have found positive feedback on our compact design, which shows a page thumbnail, one figure and its captions at a time, compared to displaying all the figures in the document. Surprisingly, we found that only one participant was familiar with similar image and text search interfaces, and those interfaces were from the data visualization domain. One possibility is that the benefits offered by images in search tools like Open-I (Demner-Fushman *et al.*, 2012) and Viziometrics (Lee *et al.*, 2017) need to be better advertised. For example, none of the 39 systems mining the COVID-19 literature (Wang and Lo, 2021) use image content.

All participants agreed that showing the caption next to the document surrogate was a significant feature. This result is consistent with recent data visualization studies highlighting the importance of text (Sultanum *et al.*, 2023). Second, filtering by modalities obtained the second highest usefulness average score (4.8), supporting the primary goal of our system. Third, displaying page thumbnails (4.4) was preferred over displaying details of subfigures or a summary of modalities. Although the page thumbnail might waste space showing illegible textual content, it provides context for other images or tables to explore next. Fourth, while participants considered showing bounding boxes (4.1) and the type of modalities found on the paper (4.1) as useful features, they expressed more neutral options toward detailing the modality of each box (3.8) or the number of images per modality found (3.7). The learned lesson is to allow the users to select the desired level of detail.

In terms of limitations, our preprocessing can be further improved. The modality prediction depends on the input image, and our segmentation tool sometimes produces images with over and under-segmentation problems. Over-segmented images created irrelevant images, while under-segmented images could contain more than one modality, negatively affecting the prediction. We leverage batch processing, and the extraction component can process two documents per minute per CPU. Alternatively, deep learning architectures could be used. Our classifiers also had minor issues predicting specific classes, e.g. when grayscale colors led to incorrect predictions of CT scans. The public availability of image modality or tagged datasets (Chen *et al.*, 2021a, b; Deng *et al.*, 2022; Jobin *et al.*, 2019; Peng *et al.*, 2021) can help by integrating a broader diversity of samples and reducing mispredictions. In addition, our online components have a 7 s latency to the front end, due to our use of an academic environment, shared virtual machine. While searching the indexes is already fast, the time required for querying the figures and database data could be improved by reducing the number of transactions, paginating indexed results and using a faster storage location.

Furthermore, our approach relies on adding the identified modality features to the search service of the Lucene server, using Lucene’s default retrieval algorithm, which is based on a vector space model and Boolean allowance supported by TF-IDF and boosting. Other approaches could combine features differently, such as creating word and image embeddings to support information retrieval beyond TF-IDF, and could have an effect on the retrieval ranking when searching. Because our focus is not on the ranking performance, we evaluate the retrieval of relevant content via precision and recall metrics.

Since the system expert evaluation, we have indexed more documents from the CORD19 collection, now searching over 43 025 documents and more than 350 000 images. Integrating this new content required about 7 h, with the majority (5.5 h) spent in the figure extraction stage. Our lab and university have adequate hardware and team resources to support expanding collections and maintain the system (Marai *et al.*, 2019). At the same time, we update our collection of labeled figures and model architectures to improve and expand our classifiers. For example, we updated our higher-modality model to consider the class ‘errors’ and to detect extraction mistakes from the segmentation pipeline, such as extracted logos from journals or blocks of text incorrectly detected as images. The trained models are available in our repository.

In conclusion, we described the design and evaluation of a search system that enhances text-based search with image-modality data. In addition, we presented a design for enhancing document surrogates with image information to improve the user experience during document retrieval. We described our solution’s different components, including a document preprocessing pipeline, image classifiers, search engine and user interface. Our evaluation shows the potential benefits of integrating image content in academic search retrieval. Feedback from domain experts further confirms the benefits of blending text and image data.

## Data Availability

The datasets were derived from sources in the public domain: CORD-19 (https://allenai.org/data/cord-19).
